# A Bayesian approach to estimate minute ventilation from heart rate during exercise for assessing environmental exposures of females

**DOI:** 10.14814/phy2.70767

**Published:** 2026-02-03

**Authors:** Gustavo Oneda, Fernando Klitzke Borszcz, Raul Würdig, Ricardo Dantas de Lucas, Rosemeri Maurici, Joseph F. Welch, Sarah Koch, Ramon Cruz

**Affiliations:** ^1^ Environmental Condition and Endurance Performance Analysis Unit (UACAMDA), Physical Effort Laboratory (LAEF), Sports Center, Department of Physical Education Federal University of Santa Catarina (UFSC) Florianopolis SC Brazil; ^2^ National Institute of Research in Sports Science and Nutrition (INPCEN) Research Center IX Florianopolis SC Brazil; ^3^ Human Performance Research Group (LAPEDH) Center for Health Sciences and Sport – Santa Catarina State University (UDESC) Florianopolis SC Brazil; ^4^ Department of Animal and Food Production Agroveterinary Sciences Center–Santa Catarina State University (UDESC) Lages SC Brazil; ^5^ Health Sciences Center (NUPAIVA) Federal University of Santa Catarina (UFSC) Florianopolis SC Brazil; ^6^ School of Sport, Exercise and Rehabilitation Sciences University of Birmingham Birmingham UK; ^7^ Department of Sport, Exercise and Health University of Basel Basel Switzerland; ^8^ Barcelona Institute for Global Health (ISGlobal) PRBB, Doctor Aiguader 88 Barcelona Spain; ^9^ CIBER Epidemiología y Salud Pública (CIBERESP) Madrid Spain

**Keywords:** environment, exposure, field‐studies, physical exercise, running

## Abstract

Estimating minute ventilation (V̇_E_) is essential for assessing the health impacts of environmental exposures during exercise field‐studies. Predictive equations using heart rate (HR) are commonly used, but overlook exercise intensity domains, and reduced accuracy is shown, particularly for females. Thus, we developed predictive equations for females' V̇_E_ based on HR responses at different exercise intensity domains using a Bayesian approach. Nineteen physically active females performed an incremental running test with breath‐by‐breath measurements of V̇_E_, metabolic rate, and HR. The first and second ventilatory thresholds were identified by measurement of the ventilatory equivalent for oxygen and carbon dioxide, respectively. The Bayesian framework showed that the model fit for estimating V̇_E_ by HR was improved when the incremental running test and its intensity domains were considered. An exponential model provided the best fit (V̇_E_ = 2.86 × exp.(0.019 × HR)) for the full incremental running test (*R* ^2^ = 0.957), whereas linear models yielded superior fits when analyzing individual moderate (V̇_E_ = −32.92 + (HR × 0.19)), heavy (V̇_E_ = −101.94 + (HR × 0.99)) and severe (V̇_E_ = −268.81 + (HR × 1.98)) exercise intensity domains (*R* ^2^ = 0.977). Accurate estimates of V̇_E_ from HR measurements must consider the exercise intensity domain and the linear regression model for better biomonitoring of human exposures.

## 1 INTRODUCTION

Measuring or estimating the total volume of air breathed every minute (i.e., minute ventilation, V̇_E_) is important to establish the interplay between human environmental exposures (such as poor air quality), exercise, and health outcomes (Cruz et al., 2020; Dons et al., 2017; Giles & Koehle, 2014; Greenwald et al., 2019; Marmett et al., 2021; Oneda et al., 2025). Specifically, during physical exercise, V̇_E_ is increased and implies higher pollutant exposure (Cruz et al., 2020, 2021; Giles & Koehle, 2014; Oneda et al., 2025), highlighting the importance of assessing V̇_E_. However, directly measuring V̇_E_ during exercise in the field is not feasible due to the need of wearing a facemask or a mouth piece over prolonged periods of time. Thus, previous studies have proposed predicting V̇_E_ based on heart rate (HR), which is easily measured through a number of methods (Cruz et al., 2020; Dons et al., 2017; Ramos et al., 2015; Zuurbier et al., 2009). Traditionally, submaximal (Zuurbier et al., 2009) and maximal (i.e., until exhaustion) (Cruz et al., 2020; Ramos et al., 2015) incremental exercise tests were performed to evaluate the relationship between HR and V̇_E_, producing a high level of agreement between the equation and direct measures of V̇_E_. However, the ventilatory response to acute exercise is intensity‐dependent; thus, using a single equation derived from an incremental exercise test could lead to inaccurate estimates of V̇_E_. For example, in humans, the ventilatory response to light or moderate exercise tends to result in slight hypercapnia, whereas the ventilatory response to heavy exercise results in hypocapnia (Welch & Mitchell, 2024), demonstrating a change in ventilatory dynamics and alterations in the relationship between V̇_E_ and metabolic rate. Conversely, the HR response to exercise is linear, with few exceptions (Laughlin, 1999).

The intensity domains of exercise are commonly demarcated by three distinct physiological phases (Binder et al., 2008; Burnley & Jones, 2007; Iannetta et al., 2022; Keir et al., 2024). More specifically, the dynamics of the rate of oxygen uptake (V̇O_2_), the rate of carbon dioxide production (V̇CO_2_), V̇_E_, and blood acid–base status are used to establish the moderate, heavy, and severe exercise intensity domains (Binder et al., 2008; Burnley & Jones, 2007). Briefly, the moderate‐intensity domain is characterized by the achievement of steady‐state V̇O_2_ and V̇_E_, and a small/minimal increase in blood lactate concentration (Iannetta et al., 2022). The heavy‐intensity domain is associated with a delayed steady‐state V̇O_2_ and V̇_E_, and a rise in blood lactate concentration before reaching a stable level (Burnley & Jones, 2007; Iannetta et al., 2022). In severe exercise, there is no physiological steady state blood lactate concentration, and V̇_E_ increases exponentially, while V̇O_2_ reaches a plateau (Binder et al., 2008; Burnley & Jones, 2007; Iannetta et al., 2022; Keir et al., 2024). These integrated physiological responses to exercise underscore the importance of considering the intensity domain when predicting changes in one variable based on another.

In addition to considering exercise intensity domains, different mathematical approaches can be utilized to improve prediction models. Bayesian inference provides posterior distributions for parameters, offering greater insights into the range of plausible values and their uncertainties, which is better than dichotomous interpretations frequently used in (“traditional”) Frequentist analysis (Kruschke & Liddell, 2018). Finally, for various societal, historical, and cultural factors, females are under‐represented in exercise science research (representing ~20% as participants), which has led to a lack of understanding in the cardiorespiratory responses of females to acute exercise (Cowley et al., 2021). Thus, we aimed to develop predictive equations for each exercise intensity domain to estimate V̇_E_ based on HR responses using a Bayesian approach for females.

## 2 MATERIALS AND METHODS

### 2.1 Participants and experimental design

The recruitment of our study was advertised through of divulgation on social media platforms and university facilities. In this regard, nineteen females (mean (± standard deviation) age 29.3 ± 7.2 years; height 162.9 ± 6.2 cm; body mass 63.1 ± 5.0 kg; body mass index 23.7 ± 1.7 kg·m^−2^; self‐reported physical activity levels 412.8 ± 133.1 min·week^−1^; self‐reported menstrual cycle duration: 27 ± 3 days), classified as “physically active,” voluntarily participated in the study. Inclusion criteria were: (a) age 18–45 years; (b) females with self‐reported regular menstrual cycle; (c) non‐smokers; (d) without neuromuscular disorders, cardiovascular, and/or respiratory dysfunction. Exclusion criteria were self‐reported: (a) musculoskeletal injury during the data collection; (b) alcohol consumption 24 h before the test; and (c) regular use of medications or anabolic steroids.

Before the incremental running test, all participants answered a physical activity and, regular menstrual cycle self‐report questionnaire and day of the menstrual cycle that the incremental running test was performed (18 ± 9 day). The participants were informed about the risks associated with the study and provided written informed consent. Body mass and height were measured, followed by an incremental running test. All participants were informed of the procedures related to the study and signed a written informed consent form prior to enrollment in the study. Ethical approval for the study was obtained from the Federal University of Santa Catarina (UFSC) Institutional Review Board (protocol number: 75076923.2.0000.0121), and all procedures were conducted in accordance with the Declaration of Helsinki.

### 2.2 Incremental running test

The incremental running test was performed on a motorized treadmill (Inbramed ATL, Porto Alegre, Brazil) using a step protocol. The treadmill incline was set at 1% gradient during the entire test. After a 5‐min warm‐up at 6 km h^−1^, speed was increased by 1 km h^−1^ every minute until volunteers reached volitional exhaustion. During the test, participants received strong verbal encouragement to continue exercising. Gas exchange/metabolic rate (V̇O_2_ and V̇CO_2_) and V̇_E_ were measured breath‐by‐breath using an automated metabolic analyzer (K5, COSMED, Rome, Italy) calibrated in accordance with the manufacturer's instructions. All data were reduced to 15‐s means, and V̇O_2_max was considered when a plateau was verified (i.e., an increase in V̇O_2_ <2.1 mL kg^−1^·min^−1^ between the last two stages) (Howley et al., 1995). When a plateau was not observed, V̇O_2_max was established based on two or more of the following secondary criteria: respiratory exchange ratio >1.1, attainment of ≥90% of the predicted maximal HR (220 bpm‐age), and RPE ≥19 (6–20 Borg's Scale). HR was recorded continuously utilizing a Polar H10 sensor chest strap device (Polar Electro Oy, Kempele, Finland). The first ventilatory threshold (VT_1_) and second ventilatory threshold (VT_2_) were determined by ventilatory equivalents for oxygen (V̇_E_/V̇O_2_) and carbon dioxide (V̇_E_/V̇CO_2_), respectively. VT_1_ was considered the initial abrupt increase in V̇_E_/V̇O_2_ without any simultaneous increase in V̇_E_/V̇CO_2_, and VT_2_ was identified as the first abrupt increase in V̇_E_/V̇CO_2_ (Binder et al., 2008) (For more details about the physiological parameters, see Table 1). Two independent and blinded investigators determined all physiological parameters.

**TABLE 1:** Mean ± SD values for absolute and relative to peak (%) physiological parameters derived from incremental running test used to estimate V̇_E_ by HR (*n* = 19).

Mean ± SD (min and max value) [percentage of the maximal/peak]					
Relative intensity	Speed (km·h^−1^)	V̇_E_ (L·min^−1^)	HR (bpm)	V̇O_2_ (mL·kg^−1^·min^−1^)	V̇O_2_ (mL·min^−1^)
VT_1_	8.1 ± 1.0 (7–10.4) [58%]	49.7 ± 12.6 (30.2–71.1) [49%]	153 ± 9.0 (138–166) [80%]	30.3 ± 5.7 (20.9–40.9) [68%]	1907.4 ± 393.8 (1380.5–2665.0) [68%]
VT_2_	11.0 ± 1.2 (9.5–13.5) [78%]	69.7 ± 16.0 (46.9–95.3) [69%]	172 ± 9.0 (157–188) [90%]	38.1 ± 6.4 (24.5–49.1) [85%]	2401.0 ± 454.9 (1614.2–3089.5) [85%]
Maximal/Peak	14.0 ± 1.5 (11.5–16.7)	100.3 ± 14.9 (72.2–122.8)	190 ± 6.0 (175–202)	44.8 ± 5.7 (34.6–55.7)	2807.2 ± 419.0 (2192.4–3478.9)

Abbreviations: bpm, beats per minute; HR, heart rate; max, maximum value; min, minimum value; SD, standard deviation; V̇E, minute ventilation; V̇O_2_, oxygen uptake; V̇O_2_max, maximal oxygen uptake; VT_1_, first ventilatory threshold; VT_2_, second ventilatory threshold.

### 2.3 Statistical analysis

The analyses were conducted within a Bayesian framework using R software (version 4.3.1) and its graphical interface RStudio (version 2023.06.1+524). The “brms” package (Bürkner, 2017) was used for analyses, which allowed the adjustment of multilevel Bayesian models using Stan software (Stan Development Team) (Gelman et al., 2015). To fit the models, we employed Markov Chain Monte Carlo techniques, specifically the No‐U‐Turn sampler. Four chains were executed in parallel for each model, consisting of 4000 interactions with a warm‐up period of 1000 iterations. We assessed the convergence of the models using Gelman–Rubin diagnostics ($\hat{R}$) (Gelman & Rubin, 1992). The relationship between V̇_E_ (L min^−1^) and HR (bpm) was assessed by two models:

#### 2.3.1 Exponential model (Equation 1)

This model consisted of a simple exponential function where V̇_E_ was the dependent variable, and HR was the independent variable. We deployed a multivariate model, allowing the intercept ($\beta_{0}$) and slope ($\beta_{1}$) to vary between participants (i.e., random effects). These random effects are represented by a between‐subjects standard deviation ($\sigma_{\beta }$). The correlation (ρ) between the coefficients was also assessed. The equation residual error was expressed as an SD ($\sigma_{\text{within}-\text{subjects}}$) and converted to a prediction interval of 95%, that represents the range within which 95% of individual future measurements are expected to fall (i.e., $\sigma_{\text{within}-\text{subjects}}\times 1.96)$.

$$\text{Likelihood}:\dot{V}E\sim \text{Normal}\left(\mu_{i},\sigma_{\text{within}-\text{subjects}}\right)$$

$$\text{Model}:\mu_{i}=\beta_{0j\left[i\right]}\times \exp^{\left(\beta_{1j\left[i\right]}\times \mathrm{HR}\right)}$$

$$\left[\begin{array}{c} \beta_{0}\\ \beta_{1} \end{array}\right]\sim \text{MVNormal}\left(\begin{array}{c} \beta_{0}\\ \beta_{1} \end{array},S\right)$$

$$S=\left[\begin{array}{cc} \sigma_{\beta_{0}} & 0\\ 0 & \sigma_{\beta_{1}} \end{array}\right]R\left[\begin{array}{cc} \sigma_{\beta_{0}} & 0\\ 0 & \sigma_{\beta_{1}} \end{array}\right]$$

$$R=\left[\begin{array}{cc} 1 & \rho \\ \rho & 1 \end{array}\right]$$

$$\text{Priors}:\beta_{0}\sim \text{Student}-t\left(3,4.2,2.5\right)$$

$$\beta_{1}\sim \text{Student}-t\left(3,0,2.5\right)$$

$$\sigma_{\text{within}-\text{subjects}},\sigma_{\beta_{0}},\text{and}\;\sigma_{\beta_{1}}\sim \text{Student}-t^{+}\left(3,0,2.5\right)$$

(1) $$R\sim \text{LKJcorr}\left(1\right)$$

where $\beta_{0}$ is the population‐level intercept and $\beta_{1}$ is the population‐level exponential coefficient for HR, respectively. The j[i], terms are the participant‐level deviations around population‐level coefficients. Those μ deviations follow a normal distribution centered on zero, including a covariance matrix. The lines following the covariance matrix are the prior distributions imposed.

#### 2.3.2 Linear model with an interaction with exercise intensity domains (Equation 2)

This model consisted of a linear function where V̇_E_ was the dependent variable, and HR, exercise intensity domains, and the interaction between HR and exercise intensity domains were the independent variables. We deployed a multivariate model, allowing the intercept ($\beta_{0}$) and slopes ($\beta_{1\;\text{to}\;5}$) to vary between subjects (i.e., random effects). These random effects are represented by a between‐subjects standard deviation ($\sigma_{\beta }$). Additionally, ρ between the coefficients, $\sigma_{\text{within}-\text{subjects}}$, and the prediction interval were assessed.

$$\text{Likelihood}:\dot{V}E\sim \text{Normal}\left(\mu_{i},\sigma_{\text{within}-\text{subjects}}\right)$$

$$\begin{array}{c} \text{Model}:\mu_{i}=\beta_{0j\left[i\right]}+\beta_{1j\left[i\right]}\left(\mathrm{HR}\right)+\beta_{2j\left[i\right]}\left({\text{Domain}}_{\text{Heavy}}\right)\\ \;+\beta_{3j\left[i\right]}\left({\text{Domain}}_{\text{Heavy}}\times \mathrm{HR}\right)+\beta_{4j\left[i\right]}\left({\text{Domain}}_{\text{Severe}}\right)\\ \;+\beta_{5j\left[i\right]}\left({\text{Domain}}_{\text{Severe}}\times \mathrm{HR}\right) \end{array}$$

$$\left[\begin{array}{c} \beta_{0}\\ \beta_{1}\\ \begin{array}{c} \beta_{2}\\ \beta_{3}\\ \begin{array}{c} \beta_{4}\\ \beta_{5} \end{array} \end{array} \end{array}\right]\sim \text{MVNormal}\left(\begin{array}{c} \beta_{0}\\ \beta_{1}\\ \begin{array}{c} \beta_{2}\\ \beta_{3}\\ \begin{array}{c} \beta_{4}\\ \beta_{5} \end{array} \end{array} \end{array},S\right)$$

$$S=\left[\begin{array}{ccc} \sigma_{\beta_{0}} & 0 & 0\;0\;\begin{array}{cc} 0 & 0 \end{array}\\ 0 & \sigma_{\beta_{1}} & \begin{array}{ccc} 0 & 0 & \begin{array}{cc} 0 & 0 \end{array} \end{array}\\ \begin{array}{c} 0\\ 0\\ \begin{array}{c} 0\\ 0 \end{array} \end{array} & \begin{array}{c} 0\\ 0\\ \begin{array}{c} 0\\ 0 \end{array} \end{array} & \begin{array}{c} \begin{array}{ccc} \sigma_{\beta_{2}} & 0 & \begin{array}{cc} 0 & 0 \end{array} \end{array}\\ \begin{array}{ccc} 0 & \sigma_{\beta_{3}} & \begin{array}{cc} 0 & 0 \end{array} \end{array}\\ \begin{array}{c} \begin{array}{ccc} 0 & 0 & \begin{array}{cc} \sigma_{\beta_{4}} & 0 \end{array} \end{array}\\ \begin{array}{ccc} 0 & 0 & \begin{array}{cc} 0 & \sigma_{\beta_{5}} \end{array} \end{array} \end{array} \end{array} \end{array}\right]R\left[\begin{array}{ccc} \sigma_{\beta_{0}} & 0 & \begin{array}{ccc} 0 & 0 & \begin{array}{cc} 0 & 0 \end{array} \end{array}\\ 0 & \sigma_{\beta_{1}} & \begin{array}{ccc} 0 & 0 & \begin{array}{cc} 0 & 0 \end{array} \end{array}\\ \begin{array}{c} 0\\ 0\\ \begin{array}{c} 0\\ 0 \end{array} \end{array} & \begin{array}{c} 0\\ 0\\ \begin{array}{c} 0\\ 0 \end{array} \end{array} & \begin{array}{c} \begin{array}{ccc} \sigma_{\beta_{2}} & 0 & \begin{array}{cc} 0 & 0 \end{array} \end{array}\\ \begin{array}{ccc} 0 & \sigma_{\beta_{3}} & \begin{array}{cc} 0 & 0 \end{array} \end{array}\\ \begin{array}{c} \begin{array}{ccc} 0 & 0 & \begin{array}{cc} \sigma_{\beta_{4}} & 0 \end{array} \end{array}\\ \begin{array}{ccc} 0 & 0 & \begin{array}{cc} 0 & \sigma_{\beta_{5}} \end{array} \end{array} \end{array} \end{array} \end{array}\right]$$

$$R=\left[\begin{array}{ccc} 1 & \rho & \begin{array}{ccc} \rho & \rho & \begin{array}{cc} \rho & \rho \end{array} \end{array}\\ \rho & 1 & \begin{array}{ccc} \rho & \rho & \begin{array}{cc} \rho & \rho \end{array} \end{array}\\ \begin{array}{c} \rho \\ \rho \\ \begin{array}{c} \rho \\ \rho \end{array} \end{array} & \begin{array}{c} \rho \\ \rho \\ \begin{array}{c} \rho \\ \rho \end{array} \end{array} & \begin{array}{ccc} \begin{array}{c} 1\\ \rho \\ \begin{array}{c} \rho \\ \rho \end{array} \end{array} & \begin{array}{c} \rho \\ 1\\ \begin{array}{c} \rho \\ \rho \end{array} \end{array} & \begin{array}{cc} \begin{array}{c} \rho \\ \rho \\ \begin{array}{c} 1\\ \rho \end{array} \end{array} & \begin{array}{c} \rho \\ \rho \\ \begin{array}{c} \rho \\ 1 \end{array} \end{array} \end{array} \end{array} \end{array}\right]$$

$$\text{Priors}:\beta_{0}\sim \text{Student}-t\left(3,64.7,24.3\right)$$

$$\beta_{1},\beta_{2},\beta_{3},\beta_{4},\text{and}\;\beta_{5}\sim \text{Student}-t\left(3,0,24.3\right)$$

$$\begin{array}{c} \sigma_{\text{within}-\text{subjects}},\sigma_{\beta_{0}},\sigma_{\beta_{1}},\sigma_{\beta_{2}},\sigma_{\beta_{3}},\sigma_{\beta_{4}},\text{and}\\ \sigma_{\beta_{5}}\sim \text{Student}-t^{+}\left(3,0,24.3\right) \end{array}$$

(2) $$R\sim \text{LKJcorr}\left(1\right)$$

where $\beta_{0}$ is the population‐level intercept and $\beta_{1}-\beta_{5}$ are the population‐level coefficients for HR, domains, and HR × domains interaction, respectively. The *j*[*i*], terms are the participant‐level deviations around population‐level coefficients. Those μ deviations follow a normal distribution centered on zero, including a covariance matrix. The lines following the covariance matrix are the prior distributions imposed. A detailed explanation of the regression procedures is provided elsewhere (Borszcz et al., 2022; McElreath, 2018).

All data in the present study are presented as posterior medians with the equal‐tailed posterior density credible interval (CrI) of 95%. The 95% CrI can be interpreted as having a 95% probability of containing the true value (Morey et al., 2016). In contrast, a 95% confidence interval within a frequentist framework is interpreted as the proportion of times that the true mean falls within the confidence interval when the same experiment is repeated (Morey et al., 2016). To determine which model provides the best estimate of pointwise out‐of‐sample prediction accuracy, we employed leave‐one‐out cross‐validation (LOO) using Expected Log Predictive Density (ELPD‐LOO) (Vehtari et al., 2017). Lastly, we evaluated the Bayesian *R* ^2^ (Bürkner, 2017).

## 3 RESULTS

**FIGURE 1:**
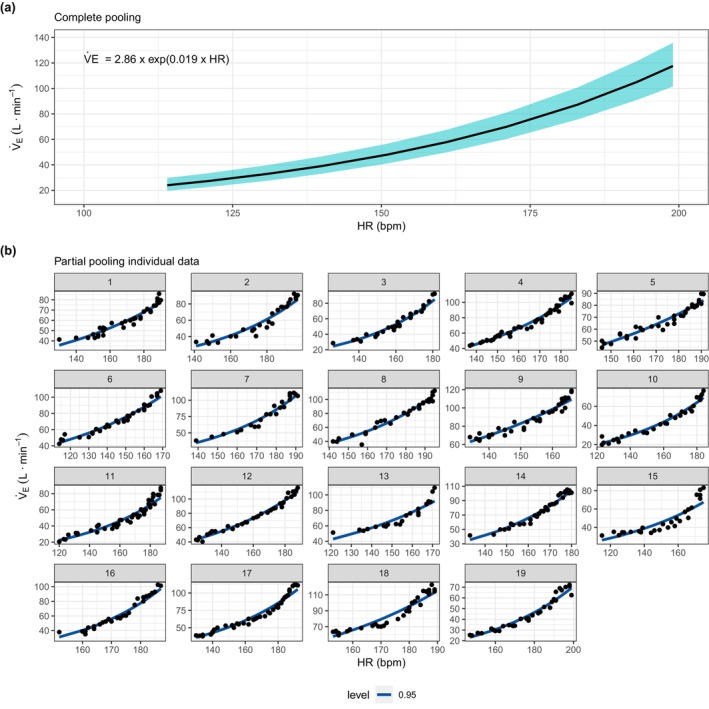
Complete pooling (population effect estimates) (a) and partial pooling (subject estimates) (b) for the exponential regression model. Data are presented as posterior medians (central lines) and 95% credible intervals. Heart rates ranged from 114 to 199 bpm (57%–100% HRpeak) among the participants in this study.

**FIGURE 2:**
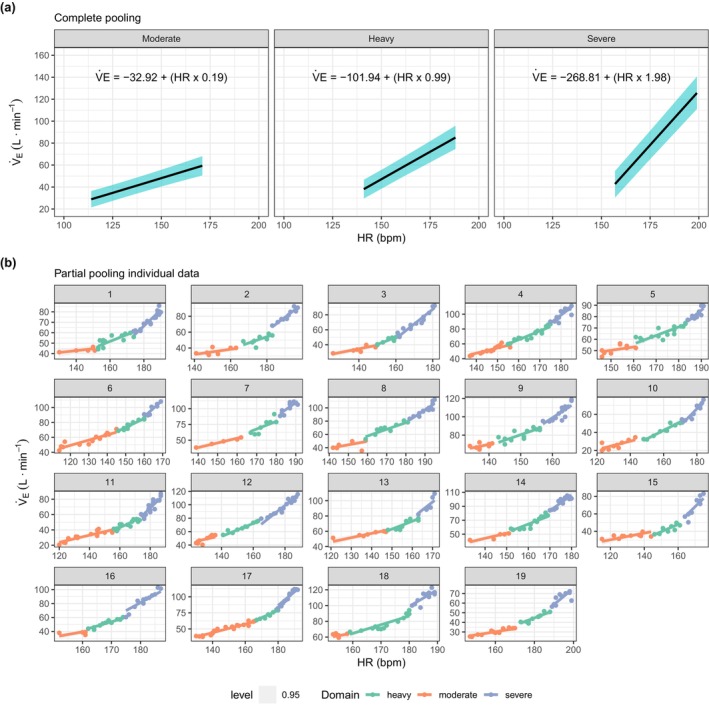
Complete pooling (population effect estimates) (a) and partial pooling (subject estimates) (b) for a linear regression model with exercise intensity domains interaction. Data are presented as posterior medians (central lines) and 95% credible intervals. Heart rates ranged from 114 to 171 bpm (57%–86% HRpeak) for moderate, 141–188 bpm (71%–94% HRpeak) for heavy, and 157–199 bpm (79%–100% HRpeak) for severe domain among the participants in this study.

The regression coefficients for the relationship between V̇_E_ and HR are presented in Table 2. Graphical representations of the regressions are provided in Figures 1 and 2 for the population estimate (complete pooling–Figures 1a and 2a) and for each individual subject (partial pooling–Figures 1b and 2b). For both models, the intercept ($\beta_{0}$) was strongly and negatively correlated with the HR coefficient ($\beta_{1}$), *ρ* = −0.79 and −0.94, respectively, suggesting that individuals with a higher $\beta_{0}$ have a lower $\beta_{1}$. All other coefficients were not correlated.

**TABLE 2:** Regression coefficients for the linear model with an interaction between exercise intensity domains and exponential model for the relationship between pulmonary ventilation (L/min) and heart rate (bpm).

Coefficients	Linear model with interaction between exercise intensity domains^a^	Traditional exponential Model^b^
Regression coefficients		
$\beta_{0}$	−32.92 [−50.54 to −16.10]	2.86 [1.87 to 4.37]
$\beta_{1}\left(\mathrm{HR}\right)$	0.54 [0.42 to 0.67]	0.019 [0.017 to 0.021]
$\beta_{2}\left({\text{Domain}}_{\text{Heavy}}\right)$	−69.02 [−88.37 to −50.80]	‐
$\beta_{3}\left({\text{Domain}}_{\text{Heavy}}\times \mathrm{HR}\right)$	0.45 [0.33 to 0.58]	‐
$\beta_{4}\left({\text{Domain}}_{\text{Severe}}\right)$	−235.89 [−264.90 to −208.20]	‐
$\beta_{5}\left({\text{Domain}}_{\text{Severe}}\times \mathrm{HR}\right)$	1.44 [1.28 to 1.61]	‐
Distributional parameters		
$\sigma_{\text{within}-\text{subjects}}$	3.50 [3.29 to 3.74]	5.01 [4.72 to 5.33]
Multilevel hyperparameters		
$\sigma_{\beta_{0}}$	25.73 [15.04 to 39.24]	7.28 [5.50 to 10.75]
$\sigma_{\beta_{1}\left(\mathrm{HR}\right)}$	0.19 [0.12 to 0.29]	0.005 [0.003 to 0.007]
$\sigma_{\beta_{2}\left({\text{Domain}}_{\text{Heavy}}\right)}$	2.88 [0.16 to 7.15]	‐
$\sigma_{\beta_{3}\left({\text{Domain}}_{\text{Heavy}}\times \mathrm{HR}\right)}$	0.02 [0.01 to 0.05]	‐
$\sigma_{\beta_{4}\left({\text{Domain}}_{\text{Severe}}\right)}$	15.39 [0.61 to 48.62]	‐
$\sigma_{\beta_{5}\left({\text{Domain}}_{\text{Severe}}\times \mathrm{HR}\right)}$	0.11 [0.02 to 0.30]	‐
$\rho \left(\beta_{0}:\beta_{1}\right)$	−0.79 [−0.93 to −0.47]	−0.94 [−0.98 to −0.86]
$\rho \left(\beta_{0}:\beta_{2}\right)$	0.15 [−0.55 to 0.74]	‐
$\rho \left(\beta_{0}:\beta_{3}\right)$	0.03 [−0.63 to 0.64]	‐
$\rho \left(\beta_{0}:\beta_{4}\right)$	0.21 [−0.49 to 0.77]	‐
$\rho \left(\beta_{0}:\beta_{5}\right)$	−0.19 [−0.73 to 0.40]	‐
$\rho \left(\beta_{1}:\beta_{2}\right)$	−0.02 [−0.66 to 0.64]	‐
$\rho \left(\beta_{1}:\beta_{3}\right)$	0.09 [−0.56 to 0.71]	‐
$\rho \left(\beta_{1}:\beta_{4}\right)$	−0.06 [−0.68 to 0.65]	‐
$\rho \left(\beta_{1}:\beta_{5}\right)$	0.31 [−0.28 to 0.76]	‐
$\rho \left(\beta_{2}:\beta_{3}\right)$	−0.13 [−0.82 to 0.67]	‐
$\rho \left(\beta_{2}:\beta_{4}\right)$	0.18 [−0.60 to 0.79]	‐
$\rho \left(\beta_{2}:\beta_{5}\right)$	0.04 [−0.67 to 0.69]	‐
$\rho \left(\beta_{3}:\beta_{4}\right)$	0.17 [−0.58 to 0.79]	‐
$\rho \left(\beta_{3}:\beta_{5}\right)$	0.15 [−0.57 to 0.75]	‐
$\rho \left(\beta_{4}:\beta_{5}\right)$	−0.42 [−0.97 to 0.50]	‐
Bayesian *R* ^2^		
*R* ^2^	0.977 [0.975 to 0.978]	0.957 [0.953 to 0.960]

*Note*: All data are presented as posterior median [95% credible intervals]. β regression coefficient, $\sigma_{\text{within}-\text{subjects}}$ within (residual/unexplained)‐subjects standard deviation, $\sigma_{\beta }$between‐subjects standard deviation, ρ correlation coefficient.

^a^ ${\dot{V}}_{E}=\beta_{0}+\beta_{1}\left(\mathrm{HR}\right)+\beta_{2}\left({\text{Domain}}_{\text{Heavy}}\right)+\beta_{3}\left({\text{Domain}}_{\text{Heavy}}\times \mathrm{HR}\right)+\beta_{4}\left({\text{Domain}}_{\text{Severe}}\right)+\beta_{5}\left({\text{Domain}}_{\text{Severe}}\times \mathrm{HR}\right)$.

^b^ ${\dot{V}}_{E}=\beta_{0}\times \exp^{\left(\beta_{1}\times \mathrm{HR}\right)}$.

The rate of increase in V̇_E_ relative to HR differed significantly across exercise intensity domains (moderate = 0.54 [0.42 to 0.67]; heavy = 0.99 [0.87–1.12]; severe = 1.98 [1.82–2.17], that is, $\beta_{1}\left(\mathrm{HR}\right)$, $\;\beta_{1}\left(\mathrm{HR}\right)+\beta_{3}\left({\text{Domain}}_{\text{Heavy}}\times \mathrm{HR}\right)$, and $\beta_{1}\left(\mathrm{HR}\right)+\beta_{5}\left({\text{Domain}}_{\text{Severe}}\times \mathrm{HR}\right)$, respectively). Pairwise comparisons revealed clear differences between all domains (moderate vs. heavy: 0.45 [0.33–0.58], probability of the effect being lower than zero [*p* < 0] = 0%; moderate vs. severe: 1.44 [1.28–1.61], *p* < 0 = 0%; heavy vs. severe: 0.99 [0.83–1.17], *p* < 0 = 0%).

The difference in predictive accuracy, as measured by ELPD‐LOO, indicates that the linear model with an interaction among exercise intensity domains is the superior model (ELPD = −1658.30) compared to the traditional exponential model (difference in ELPD ± standard error = −163.70 ± 22.10). Additionally, the *R* ^2^ value was slightly higher for the linear model (Table 2). The $\sigma_{\text{within}-\text{subjects}}$was lower for the linear model with an interaction than for the exponential model (3.5 vs. 5.0 bpm, respectively), resulting in 95% prediction intervals (i.e., representing the range within which 95% of individual future measurements are expected to fall) of ±7 and ±10 bpm, respectively.

## 4 DISCUSSION

The main finding of the present study was that predicting V̇_E_ from HR, by considering the full spectrum of each exercise intensity domain, is more accurate than using a single exponential equation modeled on data collected from a running incremental test. The accuracy of our fitted models in predicting V̇_E_ may improve the assessment in field exercise studies and thus bridge the important gap between human air pollution exposure, physical exercise, and females.

In the present study, the estimate of V̇_E_ from HR considering each exercise intensity domain showed a higher correlation (*R* ^2^ = 0.977) than applying a single equation for the running incremental test (*R* ^2^ = 0.957). Previous studies have reported high correlations using a single exponential model during a maximal bicycle ergometer incremental test with males (*R* ^2^ = 0.95) (Cruz et al., 2020) and submaximal bicycle ergometer test in males (*R* ^2^ = 0.90) and females (*R* ^2^ = 0.89) (Zuurbier et al., 2009). Another study verified a double‐linear relationship between V̇_E_ and HR, with a visually identified breakpoint corresponding to the respiratory compensation point and high correlations within each linear model (*R* ^2^ = 0.97) (Onorati et al., 2008). While these approaches represent important methodological advances, particularly in identifying this key marker, the VT_1_ was incorporated into only one of the linear models, and the relationship between V̇_E_ and HR was generally without considering the physiological differences among the three distinct exercise intensity domains.

In this sense, in the moderate exercise intensity domain, the energy supply is low, resulting in a lower fraction of oxygen (P_ET_O_2_) in the expired air, and more carbon dioxide (CO_2_) is produced. Thus, V̇O_2_, V̇CO_2_, and V̇_E_ kinetics increase linearly, as well as HR, and tend to a steady state (Binder et al., 2008). With the exercise intensity increased, in the heavy domain, the CO_2_ production increased together with a continuous rise in the P_ET_O_2_, and the fraction of oxygen (P_ET_CO_2_) tends to a steady state (Whipp et al., 1989). Centrally, receptors integrating peripheral muscle and chemoreceptors are stimulated and trigger a steeper rise in V̇_E_, while V̇O_2_ increases linearly (Binder et al., 2008; Welch & Mitchell, 2024). In the severe domain, the higher intensity results in a metabolite production exceeding the systemic elimination rate with exponential kinetics (Binder et al., 2008; Keir et al., 2024). In addition, a nonlinear rise in V̇CO_2_, with a more pronounced increase in V̇_E_, is observed. At this stage, hyperventilation fails to fully offset the H^+^ increase, resulting in a drop in P_ET_CO_2_ (Binder et al., 2008; Keir et al., 2024; Whipp et al., 1989). Accordingly, these physiological mechanisms can influence the estimates, highlighting the need for domain‐specific equations for better V̇_E_ prediction.

The V̇_E_ in environmental air pollution studies that collect data in the field/habitual settings is essential for quantifying human air pollution exposure. In fact, there is a theoretical assumption related to the anatomical/structural characteristics of females that might induce greater particle lung deposition per unit area for females than males (Kim & Hu, 1998; Liao et al., 2023). For example, current theory suggests that females have a structurally smaller laryngeal opening and a ~32% narrower tracheal cross‐sectional area than males (Eckel et al., 1994; Mann et al., 2021; Martin et al., 1987). This anatomical difference accelerates airflow during inspiration, leading to greater turbulence, known as the Venturi effect, where airflow velocity increases as it passes through a constriction due to a decrease in pressure (Kim & Hu, 1998; Rahimi‐Gorji et al., 2016). Consequently, given the elevated V̇_E_ during exercise and the heightened susceptibility to the Venturi effect, pollutants are more likely to penetrate deeper lung regions (Cruz et al., 2021; Kim & Hu, 1998). Being suggested by mathematical modeling, greater particle deposition for females at the alveolo‐bronchiolar region of the respiratory tract is likely than for males during active bicycle commuting (Oneda et al., 2025).

Some key limitations of our study must be acknowledged. The current sample presents homogenous physical fitness levels exclusively in females. Given that exercise ventilatory responses differ based on fitness, we recommend caution in extrapolating the equations to other groups. Although males and females share similar exercise ventilatory responses at relative exercise workloads, responses may differ based on absolute workload. Only one exercise testing model was considered (incremental exercise test); therefore, the model of exercise (continuous or interval training, resistance training) and the task (cycle, running, skating) may elicit different V̇_E_ responses. However, we found higher estimated V̇_E_ values than previous studies in the literature (Cruz et al., 2020; Ramos et al., 2015; Zuurbier et al., 2009), which shows that using this mathematical approach and fitting the equations by exercise intensity domain is more accurate. Finally, although HR may be influenced by potential confounders such as stress, ambient temperature, and hydration status—factors that can affect V̇_E_, HR remains a widely used and practical measure in field studies and real‐world settings to estimate exposure‐related outcomes. Moreover, in many field conditions, HR provides more meaningful physiological information than having no indicator of ventilatory demand at all. Therefore, our findings may be helpful for epidemiological studies and exercise physiologists who require evaluation of the exercise ventilatory response, human exposure from inhaled pollution dose, and biomonitoring exposure through the harmful effects of air pollution on sports and health.

## 5 CONCLUSION

We fitted different equations to estimate V̇_E_ from HR with higher predictive power during the running task. Our fitted equations, which uniquely consider exercise intensity domains, are recommended for better estimates of V̇_E_ from HR during field studies in females, and for improving the understanding of human exposure and biomonitoring the health effects.

## FUNDING INFORMATION

This research did not receive any specific grant from funding agencies in the public, commercial, or not‐for‐profit sectors.

## ETHICS STATEMENT

Ethical approval for the study was obtained from the Federal University of Santa Catarina (UFSC) Institutional Review Board (protocol number: 75076923.2.0000.0121), and all procedures were conducted in accordance with the Declaration of Helsinki.

## Data Availability

The datasets generated and/or analyzed in this study are available from the corresponding author upon reasonable request.
